# The Right to Send Private Patients to District Hospitals

**Published:** 1918-12-14

**Authors:** 


*To the Editor of* THE HOSPITAL.

### The Right to Send Private Patients to District Hospitals.

*To the Editor of* THE HOSPITAL.

SIR,—Whatever a man may think about the desirability of a State Medical Service, there is one point in the interesting articles on the subject that appeared in your columns last month that will appeal to very many. That is the suggestion that every medical man would have the right under a State Medical Service to send his cases to his district hospital, and go into the hospital to operate on them, or otherwise treat them. It is a right that would reconcile many to other drawbacks of State Service. It is very hard on a young surgeon, keen on surgery and eager to increase his skill, to have to hand over his surgical cases to the staff of a local hospital, or to have to take for himself and his patient the extra risk of disaster that must be if operation is undertaken in a private house. Even without a State Service some scheme of this sort might be worked out. If it could be, it would be greatly to the improvement of the surgical skill of the general practitioner.—Yours faithfully,

INTERESTED.

December 4, 1918.

